# Physicochemical and fertility characteristics of microalgal soil ameliorants using harvested cyanobacterial microalgal sludge from a freshwater ecosystem, Republic of Korea

**DOI:** 10.1016/j.heliyon.2022.e09700

**Published:** 2022-06-10

**Authors:** Chang Hyuk Ahn, Saeromi Lee, Jae Roh Park, Hong-Kyu Ahn, Seongsim Yoon, Kyoungphile Nam, Jin Chul Joo

**Affiliations:** a Department of Land, Water and Environment Research, Korea Institute of Civil Engineering and Building Technology, Goyang 10223, Republic of Korea; b Department of Civil and Environmental Engineering, Seoul National University, Gwanak-ro 1, Gwanak-gu, Seoul 08826, Republic of Korea; c Department of Civil and Environmental Engineering, Hanbat National University, Daejeon 34158, Republic of Korea

**Keywords:** Biofertilizer, Cyanobacterial microalgal sludge, Fertility index, Clean index, Microalgal soil ameliorant

## Abstract

The recovery and reuse strategy of cyanobacterial microalgal sludge (CyanoMS) is a novel sustainable platform that can mitigate cyanobacterial harmful algal blooms (CyanoHABs) in the freshwater system. This study aimed to assess the nutritional feasibility of harvested CyanoMS for microalgal soil ameliorants (MSAs) as efficient biofertilizers by the composting process. Most MSAs exhibited stable nutrient levels during the sequential metabolic phases for the entire period. The qualitative value of all MSAs using CyanoMS as a biofertilizer was verified by the excellent Fertility Index (FI), Clean Index (CI), and plant growth values. Also, successfully matured MSAs provided long-term support for retarded release of nutrients along the microbial transitional pathway. However, suitable CyanoMS contents of 11.7–37.6% (*w*/*w*) in MSAs were critical for efficient microbial activation and substrate inhibition. Since these results were fundamentally based on microbial transition on the CyanoMS content, optimum weight content and composting period were required. Nevertheless, MSAs were commercially applicable to high value-added crops due to their high fertilization potential and recyclable value.

## Highlights

• MSAs mixed with CyanoMS were effective biofertilizers for plants.

## Graphical abstract

**Figure:**
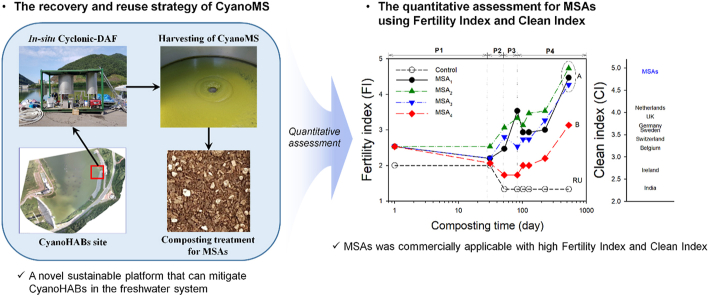

## 1 Introduction

The globally growing issues of freshwater eutrophication and cyanobacterial harmful algal blooms (CyanoHABs) are significant threats to the aquatic environment and human health. The global proliferation of CyanoHABs continues to increase with anthropogenic nutrient loading-climate synergism over 50 years (Pearl and Barnard, 2020). CyanoHABs have caused various problems to surface water quality, including color, taste/odor, sediments, and toxic substances such as microcystin and anatoxin (Pearl et al., 2011; Lee et al., 2021). To develop effective short- and long-term mitigation strategies of CyanoHABs, both proactive and responsive countermeasures (i.e., coagulation/flocculation, flotation, harvesting) of water quality management systems are required (Pearl et al., 2016). These approaches resulted in new and novel research fields that stimulate the sustainable use and circulatory system of harvested CyanoHABs. In addition, research on beneficially reusing the harvested cyanobacterial microalgal sludge (CyanoMS) has been recently highlighted (Ahn et al., 2020). These socio-technical demands have stimulated the substantial interest in beneficial reuse (i.e., biofertilizer, primary producers, antibiotics and medicines, wastewater purifier, biodiesel) of harvested CyanoMS using ecological engineering technologies and nature-based solutions (Dineshkumar et al., 2017; Gonçalves, 2021).

Historically, chemical fertilizers have been widely used in the agriculture system to increase crop yield or protect plant stress (Gonçalves, 2021). However, a recent trend is to limit the indiscriminate use of chemical fertilizers to mitigate the potential adverse effects of these agents on the aquatic ecosystem and human health. Recently, the biofertilizer has been considered one of the sustainable and environmentally-friendly solutions for these needs. In this respect, an approach using microalgal biomass as a biofertilizer would be a meaningful strategy linked to eutrophication management and resource circulation (Chojnacka et al., 2020). The harvested microalgae such as CyanoMS can be beneficially reused to produce large quantities of valuable biomass, which may facilitate higher productivity and sustainability in the fields of soil management and agriculture (Acién Fernández et al., 2018). Considering that the potential of microalgae as biofertilizers is based on their unique physicochemical properties (i.e., high nutrients, easy modification, abundant amount, and suitable combinations with other materials), biofertilizers using the harvested microalgae are perceived as an efficient source of nutrients, a renewable source of plant biostimulation, and a safe biopesticide against pathogenic bacteria (Kumar and Bera, 2020). Moreover, biofertilizers have been reported to increase the organic matter (OM) content and water-holding capacity (WHC) of soil (Uysal et al., 2015; Bhadha et al., 2017) to supply nitrogen-phosphorus-potassium (NPK) nutrients (Thompson and Meisinger, 2002; Gougoulias et al., 2018) and microelements (Gougoulias et al., 2018), and to enhance N_2_-fixation, nutrients delivery, and microbial activity (Chatterjee et al., 2017), ultimately reducing the use of chemical fertilizers (Maurya et al., 2016). Therefore, in this study, the feasibility of biofertilizers using the harvested CyanoMS was evaluated as an efficient source of nutrients and green chemicals against pathogenic bacteria to compete strongly against traditional fertilizers using animal manure.

Owing to the ability to improve soil's physicochemical properties and support plant growth, soil ameliorants using CyanoMS are promising biofertilizers (Urra et al., 2019). Additionally, soil ameliorants using CyanoMS can enhance the fundamental rhizosphere by improving both aggregated structure and aeration among soil particles (Singh et al., 2016). Thus, microalgal soil ameliorants (MSAs) can offer valuable benefits by mitigating the damages from meteorological events (i.e., floods or droughts), counteracting pest damage, enhancing the immune response in plants, securing nutrients within the soil, and assisting microbial symbiosis (Kuznetsov and Novikox, 2010). Moreover, MSAs may establish a more robust soil ecosystem in infertile regions through the early facilitation of dense plants, resulting in the prevention of both soil erosion and pollutant runoff, and hence the protection of the water quality of water resources (Kumari et al., 2013; Gonçalves, 2021).

Achieving these goals requires an economic strategy for the harvesting, recovery, and processing of CyanoMS. Traditionally, the composting strategy has been recognized as an efficient and economical technique to convert different types of organic wastes into valuable products (Ayilara et al., 2020). Consequently, successful composting processes enable the strategic hypothesis that exothermic biodegradation of CyanoMS can generate stable and mature biofertilizer with abundant nutrients and organic matter (Kuznetsov and Novikox, 2010; Kumari et al., 2013). Therefore, this study aims to produce economical and practical MSAs using composting processes from harvested CyanoMS collected from *in-situ* water treatment plants on freshwater ecosystems in the Republic of Korea. Furthermore, we analyzed the physicochemical characteristics of MSAs using CyanoMS, elucidated both fertility and safety of MSAs through the index evaluation, and investigated the microbial community structure such as composition and abundance during the composting process. Finally, the impact of the MSAs using CyanoMS on plant growth was evaluated to validate the beneficial reuse of CyanoMS by improving the soil environment and supporting plant growth.

## 2 Materials and methods

### 2.1 Harvesting of CyanoMS

**Figure 1:**
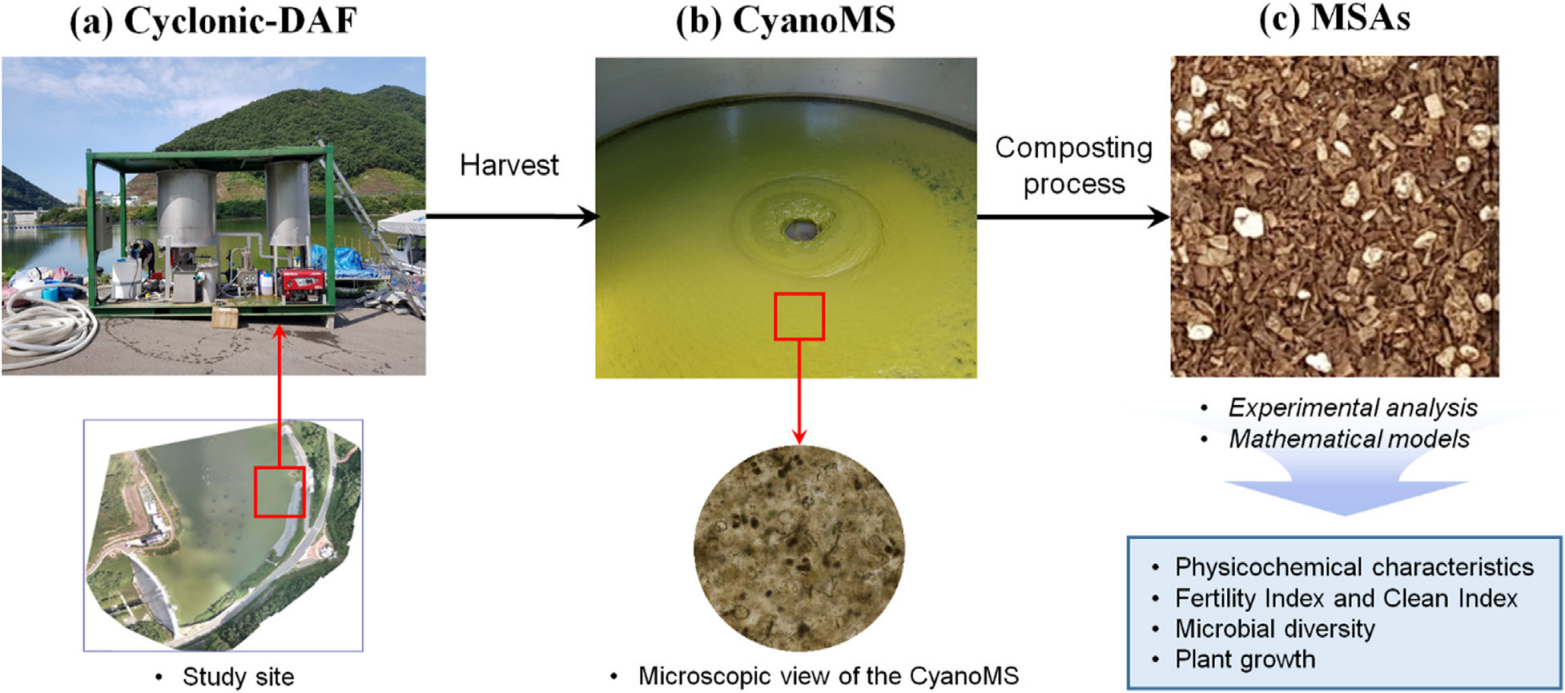
Procedures of the whole methodology to produce the MSAs using harvested CyanoMS from CyanoHABs site. (a) Cyclonic-DAF systems were located in the concentrated CyanoHABs area in the study site, (b) Appearance and microscopic view of coagulation-flocculation processes with harvested CyanoMS inside the cyclonic-DAF system. (c) Development of the MSAs using composting process and evaluated indicators in this study.

The procedures of the whole methodology covered in this study are displayed in Figure 1. The CyanoMS in this study was harvested from dam water in the Nakdong River system in August 2018. Although this newly dammed pool was completed in 2014 for water supply and flood prevention, drastic CyanoHABs have frequently occurred in the summer season due to the rapid changes (i.e., increased hydraulic retention time and nutrient accumulation) in the aquatic environment. The GPS coordinates of the *in-situ* point were 36°07′42″ N, 128°57′07″ E. The water treatment process of harvesting CyanoMS was cyclonic-dissolved air flotation (DAF) at field-scale facilities (total capacity of 240 m^3^ d^−1^ and pressure of 4.5 kgf cm^−2^). Water containing high concentrations of mixed microalgae at the edges of the water body was put inside the pilot plant. Microalgal sludge was separated using poly aluminum chloride (PAC, 10% Al_2_O_3_, 15–60 mg L^−1^) and cyclonic-DAF process with a cone-shaped sludge settling unit (Ahn et al., 2020). The initial water content of the harvested CyanoMS from the pilot plant was approximately 90% (by weight).

Microalgal parameters were analyzed by referring to the standard methods of the American Public Health Association (APHA). Collected microalgae samples using plankton net (mesh size = 60 μm) were fixed in glutaraldehyde (1–2%, JUNSEI, Japan), then identified species composition and standing crop by optical microscope (Nikon, Ni-U Co., Tokyo, Japan) in a Sedgewick-Rafter counting chamber. Chlorophyll-*a* (Chl-*a*) was analyzed by the acetone extraction method. From the preliminary results of the dam water, Chl-*a* was 235.3 μg L^−1^, and the cyanobacteria cell count was 241,000 cells mL^−1^. The composition of the microalgae species in the harvested CyanoMS was 89.3% cyanobacteria, 10.4% diatoms, and 0.3% green algae, respectively. The dominant species was *Microcystis* sp., but some *Anabaena* sp. and *Oscillatoria* sp. were also present. Since the coagulation-flocculation processes in water occurred through the interaction between various charged matters, mixtures of the microalgal cells and fine particles (i.e., silt, clay, suspended solids, extracellular organic matter, humus substances) were combined in a complex manner (Ahn et al., 2020). As a result, the surface of flocs was found to be relatively rough and dense after drying. Since fully dried CyanoMS is difficult to process due to the small amounts and low plasticity, CyanoMS with a water content of 82.6% (by weight) after natural drainage was used in this study. The detailed physicochemical characteristics of the harvested CyanoMS have been previously described (Ahn et al., 2020).

### 2.2 Composting treatment

The harvested CyanoMS was divided into four types of MSAs with different volume ratios of CyanoMS to sawdust and was subjected to aerobic composting (see Table 1). Based on the different ratios of CyanoMS to sawdust, MSAs were classified into MSA_1_ (CyanoMS, 1.5 L + sawdust, 25.5 L), MSA_2_ (CyanoMS, 3.0 L + sawdust, 24.0 L), MSA_3_ (CyanoMS, 6.0 L + sawdust, 21.0 L), and MSA_4_ (CyanoMS, 12.0 L + sawdust, 15.0 L), respectively. Also, oil cake (2.0 L), perlite (2.0 L), fermentation promoter (EM, ever-miracle, Republic of Korea) as fermented rice bran with lactic acid bacteria (0.3 L), and water (1.5 L) were added to all mixtures to ensure the appropriate initial C/N ratio (around 30), proper aeration, and vigorous microbial activity. The weight ratios (*w/w*) of CyanoMS to total weight for each initial composting samples were as follows: control (0%), MSA_1_ (11.7%), MSA_2_ (21.6%), MSA_3_ (37.6%), and MSA_4_ (59.5%). These mixtures were composted for 530 days (outside 52 days, inside 478 days) from October 2018 to March 2020 in a rotary batch reactor (H: 0.60 m × W: 0.66 m, ground height 0.34 m). The composting samples were turned over every 7–15 days to improve the aerobic microbial activity and prevent moisture deposition. The monitoring and sampling of the composting samples were performed continually at a depth of 0.3 m.

**Table 1:** Initial compositions of the control and microalgal soil ameliorants (MSAs) for the composting process.

Raw materials	Control	MSA_1_	MSA_2_	MSA_3_	MSA_4_
CyanoMS (L, *v*/*v*)	-	1.5	3.0	6.0	12.0
CyanoMS weight by fresh weight (%, *w*/*w*)	-	11.7	21.6	37.6	59.5
Sawdust (L, *v*/*v*)	27.0	25.5	24.0	21.0	15.0
Oil cake (L, *v*/*v*)	2.0	2.0	2.0	2.0	2.0
Perlite (L, *v*/*v*)	2.0	2.0	2.0	2.0	2.0
Fermentation promoter (L, *v*/*v*)	0.3	0.3	0.3	0.3	0.3
Water (L, *v*/*v*)	1.5	1.5	1.5	1.5	1.5
Total (L, *v*/*v*)	32.8	32.8	32.8	32.8	32.8

### 2.3 Determination of physicochemical characteristics

During the composting, multiple samples (500 g) were collected after 1, 31, 52, 83, 102, 127, 225, and 530 days. A portion of the collected samples was freeze-dried (FDU-8612, Hanil Scientific Medical, Republic of Korea) at -70 °C for 48 h immediately before surface and elemental analysis. Other fresh samples were used to analyze physicochemical characteristics and microbial tests. Water content was determined by drying fresh samples at 105 °C to constant weight for 24 h. The wet and dry bulk density (ρ_*wb*_, ρ_*db*_) was calculated by dividing the weight of the samples by the volume of the samples in the volumetric flask.

The total solids (TS) were measured as the dry weight remaining after oven-drying at 105 °C for 24 h. Volatile solids (VS) were measured as the change in weight of samples dried in a desiccator after ignition for 4 h at 550 °C (ash-free dry weight). Voids of volume were calculated based on the water content, dry matter, OM content, and wet bulk density (ρ_*wb*_) (Ahn et al., 2009); and the WHC was analyzed by measuring the difference in water content between 8 μm-filtered samples saturated with distilled water for two days and initial CyanoMS (Ahn et al., 2008). For the Brunauer-Emmet-Teller (BET) surface area and total pore volume analysis, the N_2_ adsorption method was used on dried samples (3Flex, Micrometrics Ins., USA), and the mercury intrusion porosimetry method was used to consider the through, closed, and blind pores of OM (MicroActive AutoPore V9600, Micrometrics Ins., USA). Additionally, salinity, HCl insoluble substances, and cation exchange capacity (CEC) were analyzed according to the fertilizer analysis methods of the Rural Development Administration of the Republic of Korea (No. 2011-46). Elemental compositions were analyzed using inductively coupled plasma optical emission spectroscopy (ICP-OES) (5100 ICP-OES, Agilent, USA) and inductively coupled plasma mass spectrometry (ICP-MS) (7900 ICP-MS, Agilent, USA) after pretreatment with mixed acids (HNO_3_:HF:H_2_O_2_ = 8:1:1, 10 mL) and microwave digestion.

### 2.4 Determination of Fertility Index and Clean Index

Physicochemical analysis was performed to evaluate the nutrients density based on the fertilizer analysis methods of the Rural Development Administration of the Republic of Korea (No. 2011-46). The analyzed parameters were as follows: OM using Walkley-Black method, total nitrogen (TN) using Kjeldahl method, ammonium-nitrogen (NH_4_^+^-N) using auto analysis method, phosphorus pentoxide (P_2_O_5_), potassium oxide (K_2_O), magnesium oxide (MgO), and calcium oxide (CaO) using the ICP method, and the organic carbon (OC) was estimated by applying a conversion coefficient to the OM.

The Fertility index (FI) was calculated to evaluate the fertilizer value of the compost products (i.e., the contribution to soil productivity). The main parameters of the FI focusing on nutritional factors included the OC, TN, P_2_O_5_, K_2_O, and C/N ratio in this study and were given by Eq. (1):

(1) $$\text{FI}=\frac{\sum_{\text{n}}^{\text{i}=1}{\text{S}}_{\text{i}}{\text{W}}_{\text{i}}}{\sum_{\text{n}}^{\text{i}=1}{\text{W}}_{\text{i}}}$$

where *S*_*i*_ is the score value of fertility (dimensionless), and *W*_*i*_ is the weighting factor of the *i*^th^ fertility value of each measured parameter.

The Clean index (CI) was calculated to evaluate the safety value of the compost products (i.e., biological stability and commercial product quality). The weighting factors in the CI for each parameter were as follows: 1 in Zn, 2 in Cu, 5 in Cd, 3 in Pb, 1 in Ni, and 3 in Cr. Cd was assigned to be the highest weighting factor because Cd can cause severe problems due to its high mammalian toxicity and bioavailability (Saha et al., 2010). On the other hand, Ni and Zn were given lower weighting factors because Ni and Zn have low or moderate mammalian toxicity and phytotoxicity potential (Lasat, 1999). The CI is given by Eq. (2), and all detailed indexations have been previously reported (Saha et al., 2010).

(2) $$\text{CI}=\frac{\sum_{\text{n}}^{\text{j}=1}{\text{S}}_{\text{j}}{\text{W}}_{\text{j}}}{\sum_{\text{n}}^{\text{j}=1}{\text{W}}_{\text{j}}}$$

where *S*_*j*_ is the score value of safety (dimensionless), and *W*_*j*_ is the weighting factor of the *j*^th^ safety value of each heavy metal.

### 2.5 Determination of microbial diversity

Microbial analysis was performed to distinguish the bacterial, filamentous fungi, actinobacteria, and *Bacillus* sp. populations in the composting samples. Samples were stored at 4 *°*C prior to the microbial analysis. The total population count was measured using the dilution plate method. For microbial cultures, egg albumin agar medium (A5503, Sigma-Aldrich, USA) was used for general bacteria and actinobacteria, rose Bengal agar medium (R1273, Sigma-Aldrich, USA) was used for filamentous fungi, and mannitol-egg yolk-polymyxin (MYP) agar medium (CM0929B, BNFKOREA, Republic of Korea) was used for *Bacillus* sp. The culture period differed depending on the growth characteristics of each microbe, and the final counts were calculated by multiplying by the dilution ratio after identification.

### 2.6 Application of MSAs on plant growth

Three experimental groups [i.e., no MSAs (i.e., commercial topsoil, control), MSAs at 250 kg 10a^−1^ (MSA_1_ to MSA_4_), and MSAs at 500 kg 10a^−1^ (2-MSA_1_ to 2-MSA_4_)] were prepared to evaluate the impact of CyanoMS on plant growth. These groups were cultivated in a greenhouse at 25 °C as a mesocosm under natural conditions for the plant growth experiment. Perilla [*Perilla frutescens* var. japonica (Hassk.) H. Hara] was used as an experimental plant for 21 days. Fresh weight and leaf conditions were monitored to evaluate both productivity and the impact of MSAs on plant growth. The collected data were statistically analyzed using R-program software (version 3.6.3).

### 2.7 Mathematical models

Both FI and nutritional indicators were analyzed according to the four parameters of the logistic (sigmoidal) equation by Eq. (3). This mathematical model in the growth analysis has been used to analyze mineralization or microbial growth during the fertilization process in previous studies (Sobratee et al., 2009; Grigatti et al., 2011). This study determined the inflection points for both the FI and nutritional indicators to predict the time for the fertility degree depending on the CyanoMS content. The logistic equation applied in this study is given as:

(3) $$\text{F}\left(\text{t}\right)={\text{F}}_{0}+\frac{{\text{F}}_{\text{m}}}{1+{\text{e}}^{\left[-\frac{\text{t}-{\text{t}}_{0}}{\text{a}}\right]}}$$

where *F(t)* represents the FI or nutritional indicators at time *t*, *F*_*0*_ represents the y intercept, *F*_*m*_ represents the maximum FI or nutritional indicators, *a* is the fertilization rate (day), *t* is the composting time (day), and *t*_*0*_ is the point of inflection (day).

The Haldane equation has been commonly used to describe substrate inhibition kinetics depending on the amount of substrate (Sonnad and Goudar, 2004; McDonald and Tipton, 2022). The Haldane equation has provided a better description of enzyme-based kinetics in microbes for a long-term period with higher substrate concentrations than the basic Michaelis-Menten equation (Koper et al., 2010). However, in cases where the substrate concentration is excessively high or potent substance inhibition (e.g., *K*_*m*_/*S* + *S*/*K*_*i*_>>1), optimization of three mediating parameters is complicated. Thus, estimation of the substrate concentration using only two mediating parameters is more appropriate (Goličnik, 2018). Two parameters (i.e., *K*_*m*_ and *K*_*i*_) determined in this study were implicitly used as apparent constants (Koper et al., 2010), and detailed equations were provided in the supplementary data [see Eq. (S1)–(S3)].

Both FI and nutritional indicators were statistically analyzed using one-way ANOVA regarding of the MSAs type, composting time, and their interactions. In addition, a Fisher's least significant difference (LSD) test was performed to evaluate the differences between the arithmetic mean values for multiple pairwise comparisons using the IBM SPSS Statistics v. 23 software package (IBM Inc., USA).

## 3 Results and discussion

### 3.1 Composting trends

**Figure 2:**
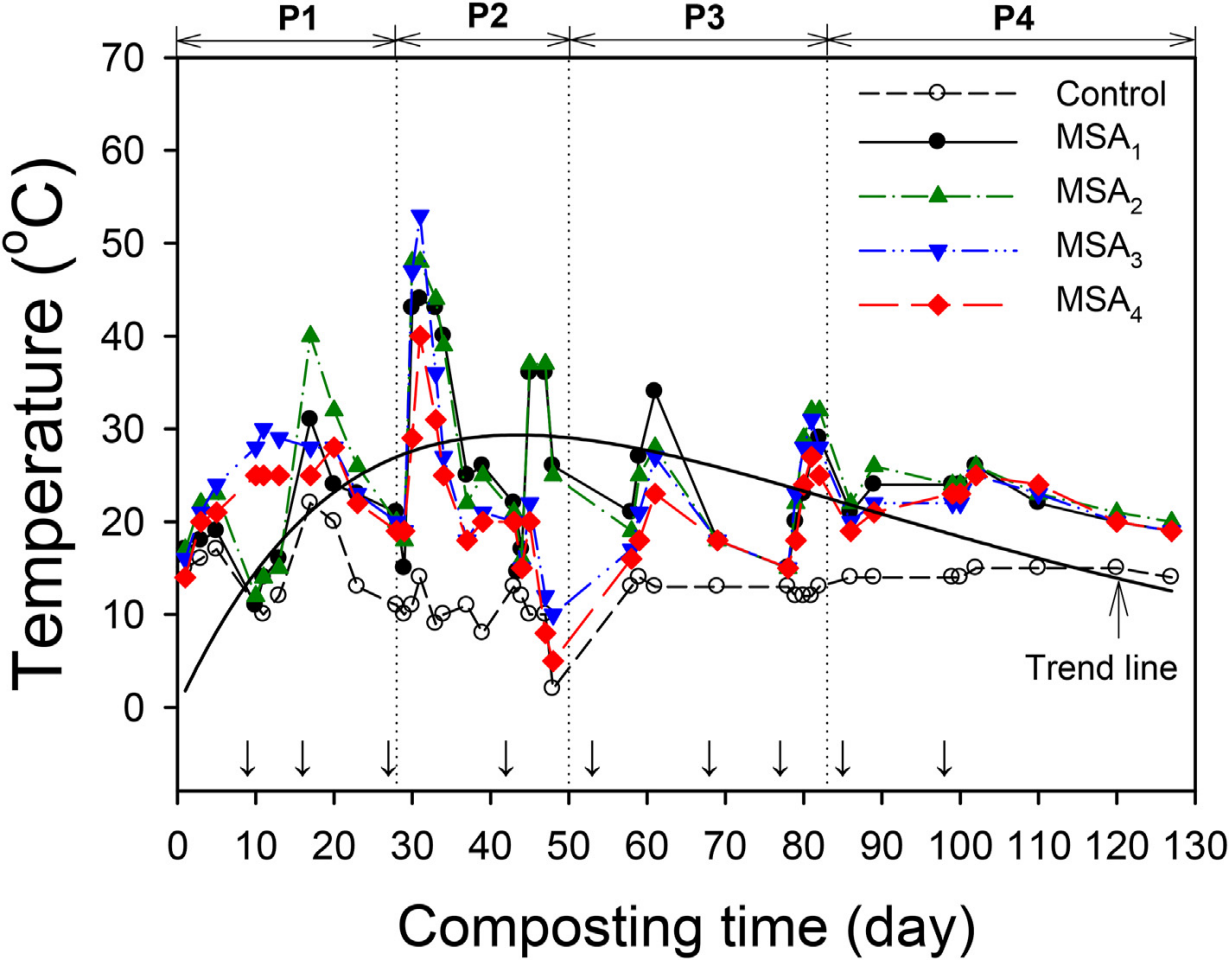
Temperature variations of the control and microalgal soil ameliorants (MSAs) during the composting process in this study. The metabolic phases included: mesophilic phase (P1), thermophilic phase (P2), cooling down phase (P3), and maturation phase (P4). The temperatures of all experimental samples were equilibrated to the external air temperatures up to 530 days (not included here), and arrow plots indicate mixing points during the composting process.

As shown in Figure 2, the temperature changes in the MSAs displayed typical variations during the composting. Based on the overall trend line in temperature variations for all MSAs, the composting process was divided into the following four phases: mesophilic phase (P1, 1–28 days) → thermophilic phase (P2, 29–50 days) → cooling down phase (P3, 51–82 days) → maturation phase (P4, >83 days). Throughout the sequential four metabolic phases, the temperature variations for all MSAs indicated a successful composting process (Chang et al., 2021). The temperature range of the MSAs was 11.0–53.0 °C, and the maximum temperature (*T*_*max*_) was 44 °C for MSA_1_, 48 °C for MSA_2_, 53 °C for MSA_3_, and 40 °C for MSA_4_, respectively. The increase in temperature of the MSAs was mainly due to the proper composting process since some of the biomass was converted to bioenergy (i.e., adenosine triphosphates or ATP); and the rest of biomass was released as heat during the aerobic metabolism by microbes (Sharma et al., 2020). For all MSAs, several temperature peaks were significant during the mixing process. Then, the final temperature was similar to the ambient air temperature (20.2 °C) after 83 days. However, the temperatures in control were lower and constant state than those in MSAs, indicating that inactive microbial metabolism occurred in control.

Based on the temperature variations, adding CyanoMS could increase the biological release of metabolic heat during composting. Also, MSAs with suitable water content and nutrient-rich environment could accelerate the degradation of OM. Consistent with these results, Han et al. (2019) reported that adding CyanoMS showed accelerated biodegradation in a humid environment. In this study, the highest temperature of MSAs during composting was approximately 53 °C, which is sufficient to obtain hygienic and mature compost products. The specific physicochemical characteristics, element compositions, changes in C/N ratio, and OM of the raw materials used in the composting process were summarized in the supplementary data (see Table S1 and Table S2).

### 3.2 Dependence of nutrients levels on the CyanoMS content

The nutrient levels classified as macro- (TN, NH_4_^+^-N, P_2_O_5_, K_2_O) and micro-nutrients (CaO, MgO) depending on the CyanoMS content with time were observed in this study. For each nutrient, the nutrient levels of the MSAs with different CyanoMS contents were diverse over time with different release behaviors, as displayed in Figure S1 in supplementary data. However, the nutrients levels considerably increased with time, irrespective of the type of nutrients. Generally, nutrients levels were accelerated to around 100 days and reached a plateau after 245 days, including an initial exponential phase, an approximately linear phase, and an asymptotic saturation phase. Similar to this study, most nutrient levels during mineralization showed an exponential or sigmoidal saturation release pattern (Grigatti et al., 2011).

In the one-way ANOVA tests, there was a statistically significant difference in the nutrient mean values between the control and the experimental groups (*p* < 0.01), suggesting that levels of various nutrients differ due to the CyanoMS content. These results indicated that CyanoMS could be used as a biostimulant to stimulate and enhance nutritional dynamics during composting. However, an excessive amount of CyanoMS in MSA_4_ suppressed the release of certain nutrients (i.e., NH_4_^+^-N and P_2_O_5_). Although CyanoMS can supply most nutrients through composting, there was a risk of suppressing nutrients release if CyanoMS content was excessively high in a specific unfavorable composting environment. These phenomena might result from inherent interactions between the unique water retention properties of cyanobacteria and microbial biodegradation activity. Consistent with this study, excessive bio-wastes (e.g., CyanoMS, sewage sludge) can suppress or slow the composting process due to unfavorable nutrients cycle conditions from inhibited biological reactions (Banegas et al., 2007; Han et al., 2014).

As is evident by Figure S1 in supplementary data, the most dynamic changes were observed in nitrogenous compounds. The initial decrease in NH_4_^+^-N observed in all MSAs was likely due to the N mineralization and full utilization of N by the microbes. During the composting, readily degradable N (high concentrations of NH_4_^+^-N) quickly decreases, while NO_3_^-^-N accumulates later by nitrifiers (Grigatti et al., 2011). In this study, MSA_4_ with a high content of CyanoMS displayed a sharp decrease in NH_4_^+^-N, whereas MSA_1_ and MSA_2_ with a relatively low content of CyanoMS displayed a significant accumulation of NH_4_^+^-N. This accumulation of NH_4_^+^-N results from complex and dynamic reactions such as nitrification, ammonia emission, ammonification, and denitrification (de Guardia et al., 2010; Manu et al., 2021).

Given the results for TN and NH_4_^+^-N in the N cycle of MSAs, MSA_3_ and MSA_4_ could be predicted to show a favorable environment for the accumulation of NO_3_^-^-N as composting progressed. Conversely, MSA_1_ and MSA_2_ could be predicted to present a successful NH_4_^+^-N accumulation with slower N-availability during composting. Another advantage of the N-dynamic derived from CyanoMS was the atmospheric N-fixation using metabolites from cyanobacteria (e.g., *Nostoc* sp., *Anabaena* sp., *Tolypothrix* sp., *Aulosira* sp.) or involvement in biogeochemical cycles under diverse environmental conditions (Chatterjee et al., 2017). Thus, the addition of CyanoMS ultimately provided both favorable conditions for a diverse living environment and substrates for ammonia-assimilating microorganisms and nitrifying bacteria. Also, these dynamic conditions by adding CyanoMS lead to the effective conversion of NH_3_–N/NH_4_^+^-N to NO_3_^-^-N, which finally enables the production of a soil ameliorant rich in various N-sources (He et al., 2020).

Among the other macro-nutrients, P_2_O_5_ and K_2_O displayed clear sigmoidal saturation curves with time, except for MSA_4_ for P_2_O_5_. These trends reveal that certain aspects of nutrients levels can finally reach an asymptotic saturation phase in the long-term composting process. However, the absence of an apparent increase in P_2_O_5_ in MSA_4_ was exceptional, probably due to the insufficient degradation or synthesis of the orthophosphates (PO_4_^3-^) in the CyanoMS (e.g., DNA, ATP, and phospholipid substances) during composting. Nevertheless, consistent with this study, many recent studies have postulated that microalgal biomass enhances the biodegradation of OM, significantly increases the levels of NPK and trace minerals, and ultimately improves the soil chemical properties with a sigmoidal saturation pattern (Abdel-Raouf et al., 2012; Renuka et al., 2016; Gougoulias et al., 2018; He et al., 2020).

Additionally, CyanoMS exhibits several nutritional properties that may contribute to MSAs development. First of all, the gradual accumulation of micro-nutrients (i.e., MgO or CaO) can be beneficial in nutritional availability to plants, distinguishing CyanoMS from other raw composting materials (e.g., animal manure and wastewater sludge) (Gougoulias et al., 2018). In addition, the accumulation of micro-nutrients in MSAs could reduce environmental stresses limiting the productivity of certain plants. Also, changes in nutrient levels by CyanoMS may be related to the physiological and structural properties of cyanobacteria. Many cyanobacteria (e.g., *Microcystis* sp.) produce different external carbohydrate structures and extracellular polymeric substances to cope with their external environment (Xu et al., 2014). These soft, multilayered, and structural substances from cyanobacteria cause rapid biodegradation during composting, leading to the early elution of readily available nutrients. Similarly, the addition of carbon sources (e.g., glucose and sucrose) can be quickly used by microbes as energy and available substances to increase the activity of microbes and nutrients assimilation. These additional substrate supplies could promote OM degradation, improve N-conversion processes, and accelerate compost maturation (Meng et al., 2021). Based on these roles, the addition of CyanoMS to compost can enhance both microbial activity and dehydrogenase enzyme activity, thereby assisting nutrients availability and maturation (El-Gamal, 2011).

### 3.3 Dependence of the FI and CI on the CyanoMS content

**Figure 3:**
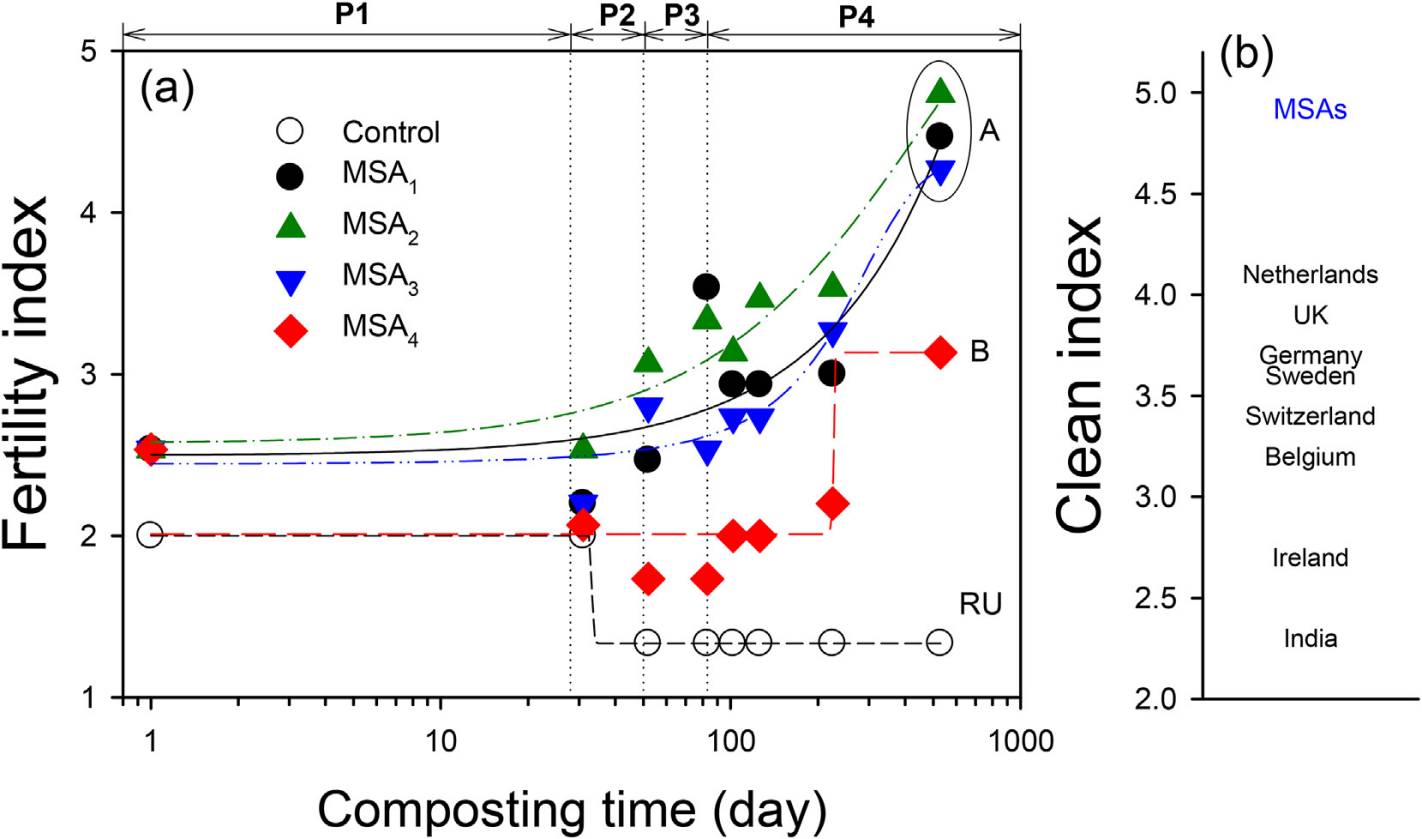
(a) The Fertility Index (FI) variations of the control and microalgal soil ameliorants (MSAs) with elapsed composting time. The metabolic phases included: mesophilic phase (P1), thermophilic phase (P2), cooling down phase (P3), and maturation phase (P4). A, B, and RU are the classification grades for the marketability in the agricultural industry. (b) The comparison of the relative legislative clean index (CI) standards between the MSAs in this study and eight different countries (data remodified based on Saha et al., 2010).

Both FI and CI are efficient indices of compost or soil quality evaluation (Saha et al., 2010; Munnaf and Mouazen, 2021) and are convenient methods with a robust quantitative assessment tool in the decision-making process. In this study, the change in the FI was described as a logistic curve reflecting microbial enzyme kinetics, as displayed in Figure 3. MSA_2_ and MSA_3_ displayed significant sigmoidal shapes (*R*^*2*^ = 0.93–0.94), MSA_1_ characterized weak sigmoidal shapes (*R*^*2*^ = 0.76), and the control and MSA_4_ did not follow a logistic curve. The control showed a low level of FI after the P2 period due to complex effects among relatively inappropriate water content, low organic carbon content, unbalanced C/N ratio, and insufficient nutrients development. Conversely, the MSAs displayed a noticeable increase in nutrients during the metabolic phases in composting of CyanoMS with the microbial community. From the results of one-way ANOVA of the FI, the hypothesis that there is no statistically significant difference of mean values of FI between the MSAs and the control was rejected within the 95% confidence interval (*p* < 0.05). Therefore, compared to the control test, MSAs have favorable composting conditions, and the growth of microbes ultimately improved fertility value, resulting in compost products with excellent quality.

As displayed in Figure 3, the overall range of the FI in this study was 1.33–4.73, and the range of the CI was 4.7–5.0. The final FI was arranged in descending order: MSA_2_ (4.73) > MSA_1_ (4.47) > MSA_3_ (4.27) > MSA_4_ (3.13) > control (1.33). Since the changes in the FI were considered as the metabolic stage of the compost products (see Figure 2), the FI showed a clear increase in the thermophilic phase (P2), after which it continued to increase throughout the cooling-down (P3) and maturation phases (P4). Since all MSAs exhibited long-term nutritional release, the MSAs improved fertility more effectively even with lower contents of CyanoMS (e.g., MSA_1_ to MSA_3_). These results indicate that sufficient improvement in soil fertility could be achieved even if a small amount of CyanoMS was applied. Moreover, continuously slow release of nutrients was observed in all MSAs' maturation phase, regardless of input CyanoMS amounts.

According to the criteria of Saha et al. (2010), MSA_1_, MSA_2_, and MSA_3_ were ‘grade A’ with high fertilizing potential and low heavy metal content, indicating the best quality (FI > 3.5, CI > 4.0; see Figure 3) used for high-value crops, such as organic farming. MSA_4_ was ‘grade B’ with medium fertilizing potential and low heavy metal content (FI of 3.1–3.5, CI > 4.0). Conversely, the control was rated as the grade of ‘restricted use’ (RU; FI < 3.1, CI was non-detect). RU refers to substances that cannot be used commercially because of their low fertilizing potential. These variations in grade validate that the MSAs evaluated in this study exhibit excellent composting performance as biofertilizers.

Regulation limits for heavy metals vary between countries (CI 2.3–4.1; e.g., Belgium, Germany, India, Ireland, Netherlands, Sweden, Switzerland, and the UK), but CI of MSAs satisfied the regulatory limits of compost for most European countries (Saha et al., 2010). Thus, the concentration of relevant heavy metals in the MSAs is safe for potential biological functions in microorganisms, phytotoxicity, and mammalian toxicity from the soil environment. Thus, CyanoMS was found to be an environmentally beneficial stimulant and the eco-toxicological safety in the soil environment could also be enhanced by using harvested microalgal biomass as an additive.

**Figure 4:**
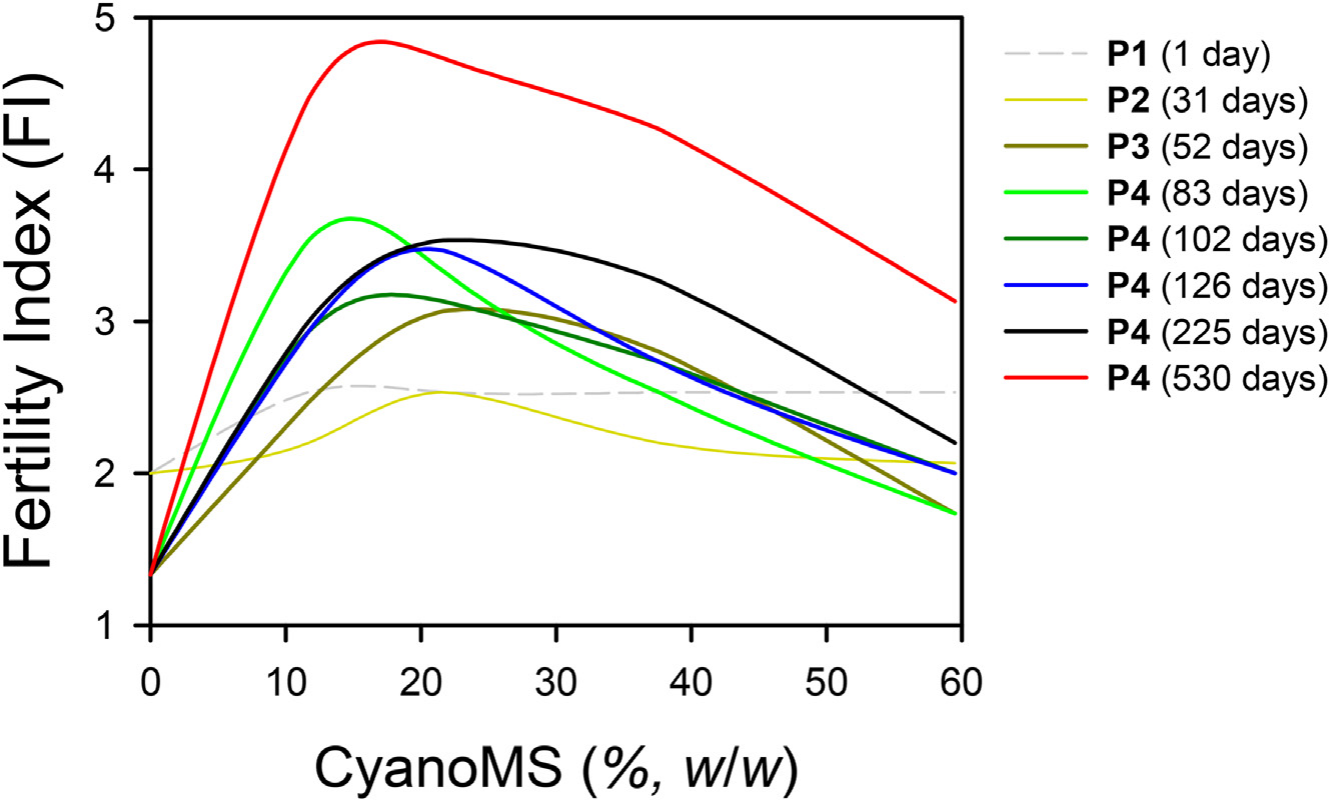
Effect of cyanobacterial microalgal sludge (CyanoMS) contents on the changes in the Fertility Index (FI) over the entire composting period. The metabolic phases included: mesophilic phase (P1), thermophilic phase (P2), cooling down phase (P3), and maturation phase (P4).

Given that the inflection points for nutrients release occurred after 102 days in the maturation phase, the release of macro- (TN, P_2_O_5_, K_2_O) and micro-nutrients (MgO and CaO) were analyzed by fittingthe Haldane equation explaining substrate inhibition. This period was analyzed because the composting process was almost complete, and there were few other disturbances. As shown in Figure 4, *S*_*max*_ was observed at approximately 20% CyanoMS content in the overall trend line. In particular, the CyanoMS content of about 15–20% suggested good fertility in analyzed composting periods. Furthermore, the trend line for the FI over time indicated considerable improvement in the cooling-down (225 days) and maturation phases (530 days). These findings revealed that optimum weight content (20%) and composting period (≥225 days) for MSAs with CyanoMS were required to improve the nutrients richness when MSAs were used as soil ameliorants.

Table 2 summarizes the results of the Haldane equation parameters for each macro- and micro-nutrients, which are components of the FI fixed between cooling and maturation phases. The mean *S*_*max*_ values were 22.2%, 11.2%, 20.6%, 20.5%, and 22.9% for TN, P_2_O_5_, K_2_O, MgO, and CaO, respectively, similar to the trend line for the FI. Both *K*_*m*_ and *K*_*i*_ were observed before and after *S*_*max*_, respectively, and P_2_O_5_ was observed to have anomalously low values. As evidenced by Figure 4 and Table 2, the Haldane equation exhibited a good fit with the reasonable coefficient of determination (*R*^*2*^ ≥ 0.88) for most variables in the inflection point, suggesting the complex relationship among microbial enzymes kinetics, substrate inhibition kinetics, and biodegradation of inhibitory substrates. Similarly, temperature, microbial populations, and microbial growth stages can be obstructively affected by abundant organic acids based on the substrate of composting microorganisms (Cheung et al., 2010). However, compared to high concentrations of organic wastes (Koper et al., 2010), CyanoMS as a substrate evaluated in this study is expected to display relatively negligible growth inhibition effects on the microbial community.

**Table 2:** Summary of mediating parameters of Haldane equation in terms of macro-nutrients (TN, P_2_O_5_, K_2_O) and micro-nutrients (MgO, CaO) concentrations with different cyanobacterial microalgal sludge (CyanoMS) contents from the cooling down phase to maturation phase (102–530 days).

Parameters	Days	*F*_*m*_	*S*_*max*_	*K*_*m*_	*K*_*i*_	*R*^*2*^	*P*
Macro-nutrients							
TN	102	8.59	21.78	6.60	43.57	0.88	0.019
	127	8.36	22.02	6.83	44.05	N.D.	N.D.
	530	14.65	22.79	4.07	45.58	N.D.	N.D.
Average ±S.D.		10.5 ± 2.9	22.2 ± 0.4	5.8 ± 1.2	44.4 ± 0.9	-	-
P_2_O_5_	102	5.07	10.51	2.72	21.02	0.91	0.012
	127	5.49	9.67	2.09	19.34	0.76	0.053
	530	6.86	13.55	3.34	27.09	0.52	0.168
Average ±S.D.		5.8 ± 0.8	11.2 ± 1.7	2.7 ± 0.5	22.5 ± 3.3	-	-
K_2_O	102	4.09	20.35	8.13	40.70	0.96	0.003
	127	4.18	21.69	8.76	43.38	0.18	0.472
	530	7.57	19.72	6.08	39.44	0.71	0.074
Average ±S.D.		5.3 ± 1.6	20.6 ± 0.8	7.7 ± 1.1	41.2 ± 1.6	-	-

Micro-nutrients							
MgO	102	0.46	16.64	8.09	33.28	0.95	0.005
	127	0.65	26.46	12.90	52.91	0.97	0.002
	530	1.53	18.51	8.49	37.03	0.97	0.002
Average ±S.D.		0.9 ± 0.5	20.5 ± 4.3	9.8 ± 2.2	41.1 ± 8.5	-	-
CaO	102	0.35	20.31	9.98	40.63	0.89	0.016
	127	0.39	29.19	14.40	58.39	N.D.	N.D.
	530	0.76	19.15	9.20	38.30	0.69	0.080
Average ±S.D.		0.5 ± 0.2	22.9 ± 4.5	11.2 ± 2.3	45.8 ± 9.0	-	-

### 3.4 Dependence of microbial transition on the CyanoMS content

**Figure 5:**
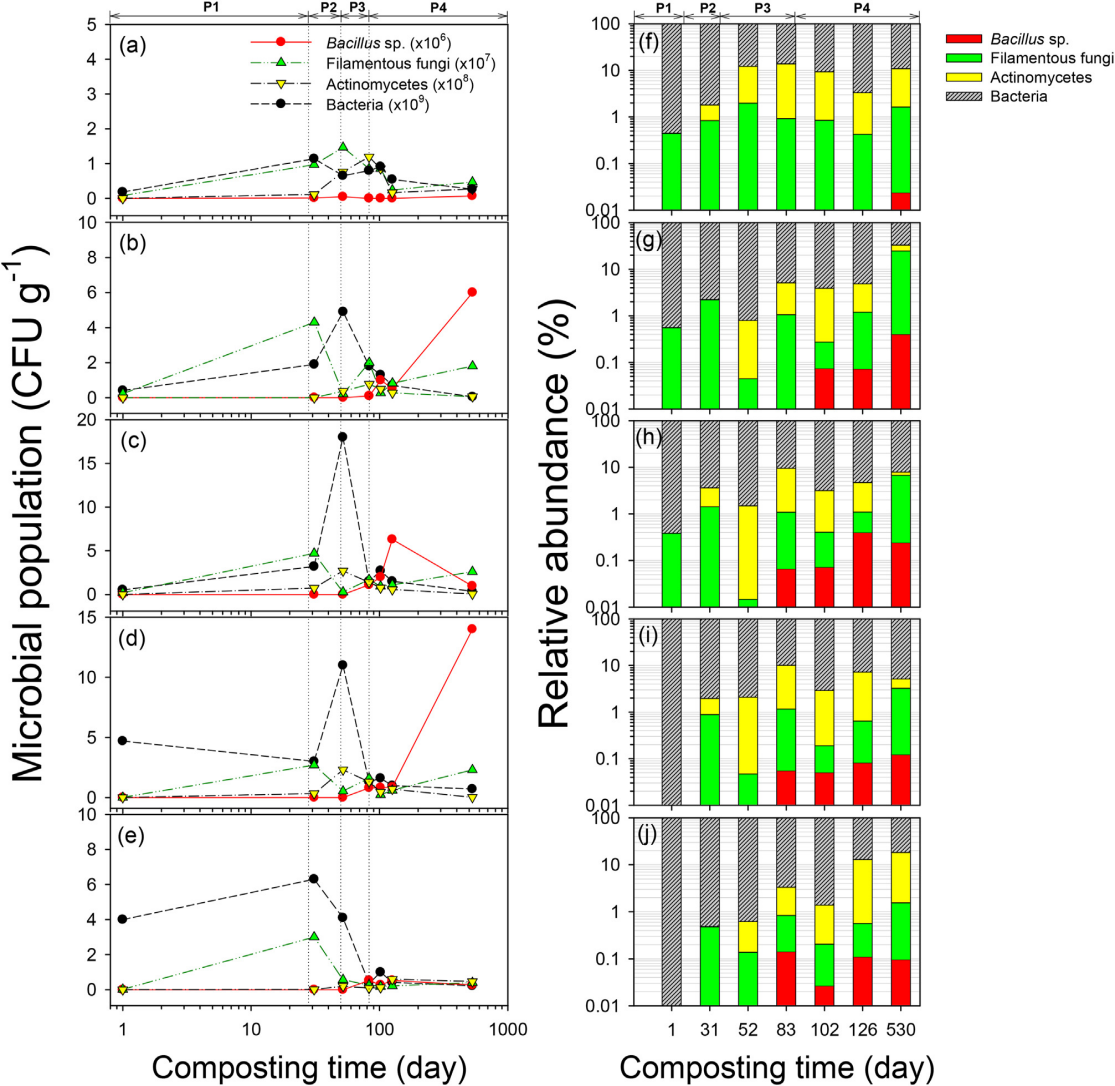
Changes in the microbial populations (a–e) and relative abundance (f–j) of four major microbial communities for control and microalgal soil ameliorants (MSAs) over the entire composting period. (a, f) Control, (b, g) MSA_1_, (c, h) MSA_2_, (d, i) MSA_3_, and (e, j) MSA_4_. The metabolic phases included: mesophilic phase (P1), thermophilic phase (P2), cooling down phase (P3), and maturation phase (P4).

Figure 5 displays how the CyanoMS content affected the total microbial population during composting. The total microbial population increased rapidly during the exponential growth phase and decreased in the order of MSA_2_ > MSA_3_ > MSA_1_ > MSA_4_ > control. The overall change in the microbial community followed the transitional pathway depending on the metabolic stage of the composting phases: mesophilic phase (bacteria, filamentous fungi) → thermophilic phase (actinomycetes, bacteria) → cooling down phase (actinomycetes, *Bacillus* sp.) → maturation phase (*Bacillus* sp.); which was consistent with the results for the FI and nutrients release (see Figure S2 in supplementary data).

The transition of microbial communities during the entire composting period provided fundamentally significant development in the fertility of the MSAs. First, both bacteria and filamentous fungi were involved in the mesophilic phase. Initial carbohydrates were degraded more rapidly than lignin, and the OM was degraded non-selectively by bacteria and filamentous fungi during this period. Next, the mesophilic phase was replaced with the thermophilic phase. During this time, the microbial community contained actinomycetes alongside bacteria and filamentous fungi. These microorganisms were typically involved in the degradation of macromolecular fibers (i.e., cellulose, hemicellulose, and pectin) after the rapid degradation of soluble compounds. Due to elevated temperature (50–80 °C) during the thermophilic phase, non-spore-forming pathogenic bacteria (e.g., *Salmonella*, *E. coli*) were not observed in any samples since pathogenic microorganisms are effectively inactivated with high temperatures (50–80 °C) (Barthod et al., 2018). In the cooling-down phase, both degradation rate and temperature decreased. As a result, non-degradable OM, such as lignin, dominated the residue substrate. Most microbes endured a brief stationary phase and death phase in this period, but some species, such as *Bacillus* sp., thrived. This *Bacillus* sp. community was primarily detected in the later composting stages for MSAs containing CyanoMS, but the increasing rate was also associated with high FI and maturity. Moreover, *Bacillus* sp. in MSAs can inhibit the growth of phytopathogens and fungi in well-matured compost involving intrinsic circadian rhythms. Therefore, these microbial communities in MSAs can act as biological pesticides (Zaccardelli et al., 2020; Eelderink-Chen et al., 2021). Finally, a prolonged stationary phase was observed during maturation. During this period, most microbial growth ceases, but some cells can maintain their metabolic activity. Also, certain microorganisms build survival strategies such as protein synthesis, which are necessary even under adverse conditions with insufficient substrate environments, through gene expression such as stationary phase promoters (Jaishankar and Srivastava, 2017). The appearance of *Bacillus* sp. in the maturation period in MSAs suggests that final products have the potential to continuously produce nutrients or secondary metabolic products through high enzyme activity to degrade raw waste materials (Simarmata et al., 2021). During this time, microbial consortia would degrade intracellularly stored substances by endogenous metabolism and form new constituent units and energy monomers in a stable state. Conversion of intracellular regulatory mechanisms by whole microbial transitional pathway verifies the ability to support the retarded release of nutrients during the maturation phase.

The microbial population changes were complexly involved in the characteristics of raw materials composition, utilization of the additive, and the physicochemical quality of the final biofertilizers (Muscolo et al., 2018). Thus, in this study, the CyanoMS content of 11.7–37.6% by weight was critical for efficient microbial activation and composition. Moreover, the higher than 37.6% of CyanoMS content matched the substrate inhibition found in the FI (see Figure 4). For all MSAs, we believe that aerobic and non-photosynthetic microbes used in the composting process can produce various physiologically active substances, including hydrolytic and thrombolytic enzymes, functional peptides, and macromolecular mucilage. The close relationships between microbial activity and fertility evolution can be explained by the analogous nature of the microbial growth phases and metabolic stages of the composting phases.

### 3.5 Dependence of plant growth on the CyanoMS content

**Figure 6:**
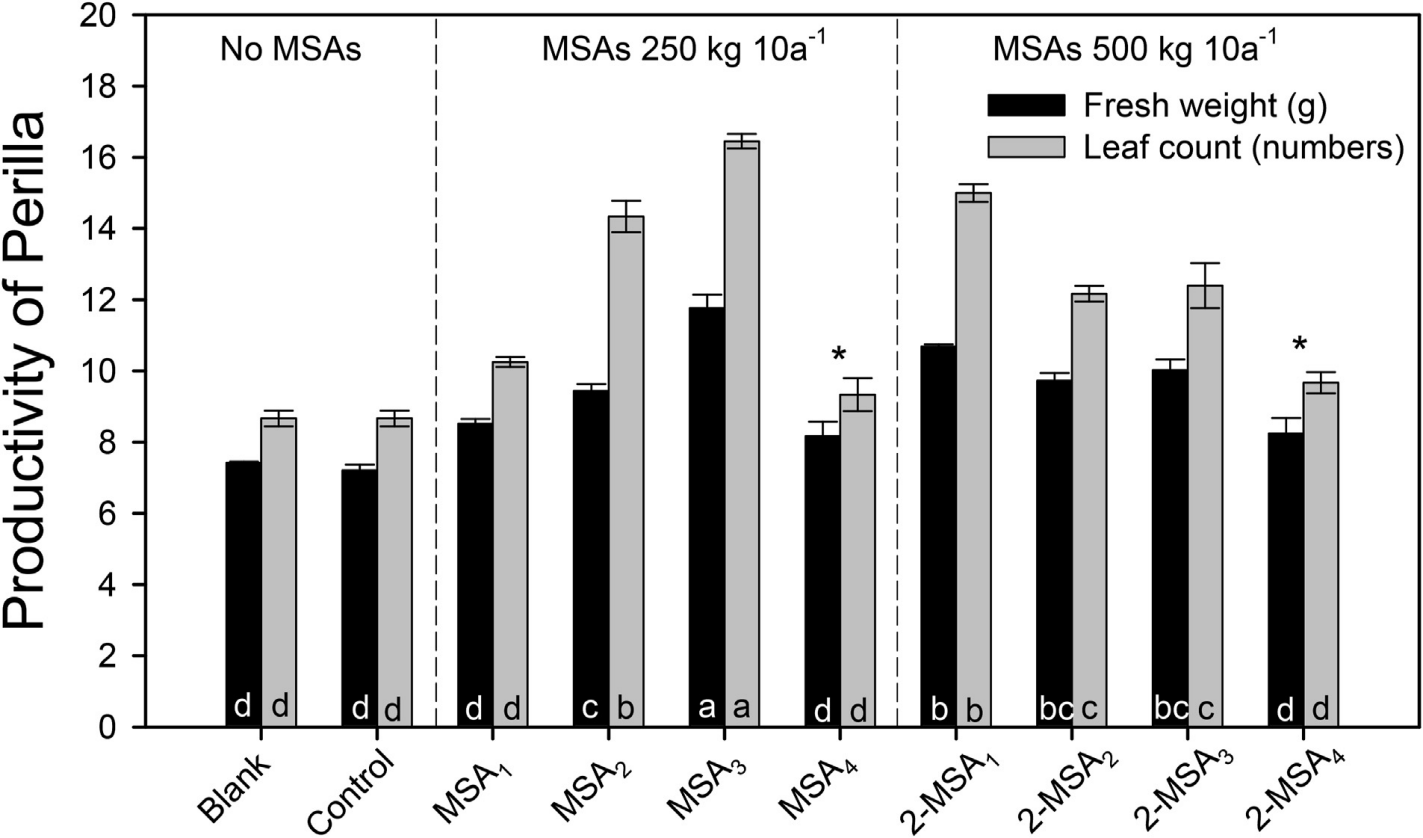
Comparison of Perilla productivity (fresh weight and leaf count) for the control and microalgal soil ameliorants (MSAs) at the composting time of 530 days. Note that error bars represent the standard error, and asterisks indicate harmful effects perceived in small parts during the entire period of plant growth.

The impact of CyanoMS content on plant growth is displayed in Figure 6. Perilla productivity was greater for MSAs with CyanoMS (i.e., maximum fresh weight 63.3%, leaf count 89.9% increase) than without CyanoMS. These positive outcomes for biomass productivity indicate the nutritional value of CyanoMS in the Perilla growth. Also, the fertility impacts of the MSAs primarily supported vegetative development in the early stages by providing a transition foundation for reproductive growth. Whereas the increase in fresh weight and leaf count was greater in the 250 kg 10a^−1^ MSA_3_ group, that of both control using commercial topsoil and blank with no CyanoMS group were not significant. For example, the fresh weight of Perilla decreases in the order of MSA_3_ > 2-MSA_1_ > 2-MSA_3_ > 2-MSA_2_ > MSA_2_ > MSA_1_ > 2-MSA_4_ > MSA_4_ > control > blank, and the leaf count of Perilla decreases in the order of MSA_3_ > 2-MSA_1_ > MSA_2_ > 2-MSA_3_ > 2-MSA_2_ > MSA_1_ > 2-MSA_4_ > MSA_4_ > control = blank (one-way ANOVA, Duncan test, *α* = 0.01). These statistical results suggest that MSAs are potential soil ameliorants supporting plant growth, although a minor growth inhibition effect was only observed in MSA_4_ and 2-MSA_4_ with the highest CyanoMS content.

One unique finding from this study was that the FI and plant growth results were not always consistent. For example, the highest FI was estimated in MSA_2_ (see Figure 3), whereas the maximum growth of Perilla was observed in MSA_3_ (see Figure 6). These results could be due to the variation of physiological abilities and particular nutritional demands depending on the specific growth stage of a particular plant. Similarly, many researchers reported that plant growth could differ depending on the concentration of required inorganic elements (Sun et al., 2020), environmental conditions (Madhavi et al., 2021), the growth stages (Nakanwagi et al., 2020), nutrients absorption (Kong et al., 2016), and the state of soil properties (Abdul Khalil et al., 2015). Also, previous studies reported that nutrients concentration and fertilization ratio were essential for strategic plant growth (Deng et al., 2019; Ann et al., 2020). Therefore, the different plant productivity in terrestrial ecosystems potentially depends on the fertility grade and the specific nutrient ratio with complex soil environment (Peng et al., 2017).

According to the previous study, green chemicals (e.g., fatty acids, phenolic substances) abundant in CyanoMS can positively affect physiological metabolism related to plant life cycles, such as antioxidant, biostimulant, and pesticide effects (Ahn et al., 2020). In this regard, MSAs successfully contain a diverse microbial community and therefore have a high probability of natural symbiosis in the rhizosphere by producing valuable secondary metabolites. Thus, MSAs could be potentially beneficial in various developmental phases of plants, including sprouting, division, flowering, differentiation, and ripening. Moreover, the abundant OM in the MSAs formed through aerobic fermentation can support continuous nutritional supply to plants via linkage with various valuable green chemicals from the CyanoMS.

## 4 Conclusions

This study found that adding appropriate CyanoMS to the composting process accelerated the degradation of OM, and enhanced the suitable water content and the nutrient-rich environment with the thermophilic phase. Also, the addition of CyanoMS to the composting process provided favorable conditions (the effective conversion of NH_3_-N/NH_4_^+^-N to NO_3_^-^-N) for ammonia-assimilating microorganisms and nitrifying bacteria. Furthermore, the gradual accumulation of micro-nutrients (i.e., MgO or CaO) can be beneficial in nutritional availability to plants, clearly distinguishing CyanoMS from other raw composting materials (e.g., animal manure and wastewater sludge).

The MSAs prepared in this study were proved to be a commercial soil ameliorant that was nutritive excellent and ecologically safe. Regardless of the input amount of CyanoMS, all MSAs could achieve sufficient improvement in fertility capacity. Also, both continuous and slow release of nutrients were observed in the long-term maturation phase. Furthermore, all MSAs were graded A and B with high fertilizing potential and low heavy metal content used for high-value crops. Conversely, the control was rated as restricted use, validating that the MSAs evaluated in this study exhibit excellent composting performance as a biofertilizer.

CyanoMS content of 11.7–37.6% by weight was critical for efficient microbial activation and supported the qualitative performance of the fertile MSAs with the optimum composting period. Besides, MSAs can significantly assist early developmental phases through continuous nutritional supply to plants via linkage with various nutrients and valuable green chemicals. Consequently, CyanoMS was an environmentally-beneficial biostimulant, and MSAs could enhance the eco-toxicological safety in the soil environment using harvested microalgal biomass as an additive. The findings of this study are expected to contribute to the replacement of the commercial fertilizers with harvested CyanoMS to reduce the excessive use of chemical fertilizers and sustain the health of agricultural soil ecosystems. Although this study focused on nutrient development according to the application of CyanoMS, information on critical microbial mechanisms related to substrate inhibition was limited. Further study is warranted to evaluate ecotoxicity based on microbial kinetics under various plant growth stages.

## Declarations

### Author contribution statement

Chang Hyuk Ahn: Conceived and designed the experiments; Performed the experiments; Analyzed and interpreted the data; Contributed reagents, materials, analysis tools or data; Wrote the paper.

Saeromi Lee: Performed the experiments; Contributed reagents, materials, analysis tools or data.

Jae Roh Park, Hong-Kyu Ahn, Kyoungphile Nam: Analyzed and interpreted the data.

Seongsim Yoon: Performed the experiments.

Jin Chul Joo: Analyzed and interpreted the data; Wrote the paper.

### Funding statement

This work was supported by the Major Project of the Korea Institute of Civil Engineering and Building Technology (2021-0207, 2022-0194).

### Data availability statement

Data included in article/supplementary material/referenced in article.

### Declaration of interests statement

The authors declare no conflict of interest.

### Additional information

No additional information is available for this paper.

## Appendix A Supplementary data

Supplement _Fig1 Supplement _Fig1
