# Supplementary material for: Physicochemical and fertility characteristics of microalgal soil ameliorants using harvested cyanobacterial microalgal sludge from a freshwater ecosystem, Republic of Korea
Source: Heliyon. 2022 Jun 10;8(6):e09700. doi: 10.1016/j.heliyon.2022.e09700 (PMC9233212; doi:10.1016/j.heliyon.2022.e09700)
Supplement: Supplement _Fig1 [file mmc1.docx]

Supplementary Data

**Physicochemical and fertility characteristics of microalgal soil ameliorants using harvested cyanobacterial microalgal sludge from a freshwater ecosystem, Republic of Korea**

Chang Hyuk Ahn*^a,b*^*, Saeromi Lee*^a^*, Jae Roh Park*^a^*, Hong-Kyu Ahn*^a^*, Seongsim Yoon*^a^*, Kyoungphile Nam*^b^*, Jin Chul Joo*^c*^*

*^a^*Department of Land, Water and Environment Research, Korea Institute of Civil Engineering and Building Technology, Goyang 10223, Republic of Korea

*^b^*Department of Civil and Environmental Engineering, Seoul National University, Gwanak-ro 1, Gwanak-gu, Seoul 08826, Republic of Korea

*^c^*Department of Civil and Environmental Engineering, Hanbat National University, Daejeon 34158, Republic of Korea

* Corresponding Author:

Tel: +82-42-821-1264, E-mail: [jincjoo@hanbat.ac.kr](mailto:jincjoo@hanbat.ac.kr)

Tel: +82-31-910-0743, E-mail: [chahn@kict.re.kr](mailto:chahn@kict.re.kr)

Information of Supplementary Data

12 Pages; 2 Tables; 2 Figures

**Parameters and weighting factors for Fertility Index (FI) calculation**

Considering the functions of each parameter, the weighting factors for the FI were set as follows: 5 in OC, 3 in TN, 3 in P_2_O_5_, 1 in K_2_O, and 3 in C:N ratio. C is important for plant biomass because it regulates several soil productivity parameters (e.g., WHC, porosity, soil structure, plant nutrients reserve pool, and promotes biological activity). C-rich fertilization not only shows higher residence time due to the formation of clay-humus complexes but can also mitigate climate change by contributing to C-storage. For this reason, OC has the highest weight factor (Saha et al., 2010). Although all the three major nutrients (TN, P_2_O_5_, and K_2_O) are essential for higher crop productivity, they were assigned to be different weighting factors based on their functional importance and prevalence of deficiency in soils (Saha et al., 2010).

| **Expression of FI in accordance with Haldane equation**  $F= \frac{F_{m}S}{K_{m}+S+ \frac{S^{2}}{K_{i}}} = \frac{F_{m}}{1+ \frac{K_{m}}{S} + \frac{S}{K_{i}}} = \frac{F_{m}K_{i}S}{K_{m}K_{i}+K_{i}S+S^{2}}$; | (S1) |
| --- | --- |
| $F= \frac{F_{m}S}{K_{m}+ \frac{S^{2}}{K_{i}}} = \frac{F_{m}K_{i}S}{K_{m}K_{i}+S^{2}} = \frac{k_{1}S}{k_{2}+S^{2}}$; | (S2) |
| $S_{\max}=\sqrt{K_{m}K_{i}}$; | (S3) |

where *F* represents the FI or nutritional indicators in **Eq. S1**, *F_m_* represents the maximum FI or nutritional indicators of the reaction, *S* represents the CyanoMS weight (%, *w*/*w*) as the major substrate, *K_m_* is equal to the minimum CyanoMS weight that produces a rate of 1/2 the *F_m_* and represents the dissociation constant for the substrate complex, *K_i_* is equal to the maximum CyanoMS weight that produces a rate of 1/2 the *F_m_* and represents the inhibition constant for the substrate complex, and *S_max_* represents the CyanoMS weight with maximum FI or nutritional indicators of the reaction. Both *k_1_* ( = F*_m_K_i_*) and *k_2_* ( = *K_m_K_i_* or *K_m_K_i_* + *K_i_S*) in **Eq. S2** are not parameters in the original Haldane equation, but are intrinsic constants used to simplify **Eq. S3** (Goličnik, 2018).

**Physicochemical characteristics and element compositions**

The physicochemical characteristics and element compositions of the raw materials, as well as the final products were summarized in **Table S1**. The raw material (i.e., CyanoMS) had high water content and WHC and low VS content and porosity with various ranges of the specific surface area; whereas the additives (i.e., sawdust, oilcake, and perlite) had low water content and WHC, and high VS content and porosity with relatively low specific surface area. Therefore, the additives can complement the properties of CyanoMS and improve the composting conditions by increasing TS content with reduced water content.

As shown in **Table S1**, the MSAs clearly had favorable physicochemical properties as soil ameliorants with a suitable level of moisture (mean 40.3–55.2%), pH (mean 6.4–7.1), high VS (mean 61.6–76.9%), high V_a_ (mean 76.0–87.2%), and improved CEC (mean, 40.80–49.40 cmol kg^-1^). In particular, the MSAs using CyanoMS were characterized by relatively high OM content and high void properties except for MSA_4_, and these physicochemical properties of soil ameliorants using exogenous organic matter (i.e. CyanoMS) are similar to those of soil ameliorants using soil organic matter (Zaffar and Lu, 2015). Since the CyanoMS in this study was harvested from *in-situ* water treatment plants in freshwater ecosystem, the CyanoMS contained a large quantity of fine particles including mixed culture of freshwater microalgal species. This complex mixture in CyanoMS may be suitable in the improvement of the physicochemical properties of soil through the development of aggregated structures of diverse particles (i.e., silt, clay, suspended solids, extracellular organic matter, humus substances, etc.) with various chemical components. Thus, the addition of these CyanoMS can increase the voids (V_v_) and air-filled porosity (V_a_) in the final soil ameliorants, thereby improving the aerobic conditions of the mixture (i.e., MSAs) and providing the essential nutrients available for plant growth.

Based on the results of element composition analysis summarized in **Table S1**, the chemical properties of the MSAs were found to be suitable as a soil ameliorant. The chemical composition of CyanoMS was different from that of single cultures of microalgal biomass, such as green algae (GA in **Table S1**) as *Chlorella* sp. or blue green algae (BGA in **Table S1**) as *Spirulina* sp. Compared to the pure cultures of microalgal biomass, relatively low N and P, high C/N ratio, high concentration of cations (Al, Mg, Ca, and K) of harvested CyanoMS are mainly attributed to coagulation-flocculation processes in the water treatment plant. As the destabilization of microalgal cells in water with coagulant-flocculant-floatation processes enables formation of CyanoMS facilitating various solid networks in water, relatively low N and P and high concentration of cations resulted as compared to the pure cultures of microalgal biomass.

As is indicated in **Table S1**, CyanoMS contains abundant macro-nutrients (e.g., N, P, K, S, Ca, and Mg) supporting plant growth, and hence compost products from CyanoMS can provide rich macro-nutrients to plants. However, some heavy metals and toxic substances accumulated in CyanoMS could be considered as latent risk factors. In this study, the heavy metal content of both CyanoMS and MSAs was much lower than that of sewage sludge, and also satisfied international criteria (Brinton, 2000). Although the toxic substances produced by cyanobacteria (e.g., cyanotoxins) were not intensively investigated in this study, they were reported to be easily degraded after composting under the detection limits (Huang et al., 2014). Therefore, MSAs after long-term maturation are safe soil ameliorants.

As also displayed in **Table S2**, the change in C/N ratio of all MSAs with elapsed time showed a similar trend to that of OM. The C/N ratio and OM content decreased rapidly up to 100 days, and then slowly reached a plateau around 530 days. Considering that an appropriate C/N ratio is around 30 during the initial period and less than 20 in the final product (Sánchez et al., 2017), C/N ratios of all MSAs except for control were within the appropriate ranges. Also, the OM content of all MSAs was 13.1–27.9% which is sufficient for compost. However, the control did not show sufficient decomposition of OM. These appropriate C/N ratios and the abundant OM content in the MSAs were the evidence of successful maturation resulting in circulation of nutrients, improvement of physicochemical function, and formation of a suitable living environment for diverse microorganisms in soil when the MSAs were used as soil ameliorants. Based on these results, CyanoMS supported the appropriate levels of C/N ratios and OM content throughout the composting process.

Consistent with previous studies (Qian et al., 2014; Han et al., 2019) postulating that cyanobacteria biomass possessing a structure with high viscosity needs a long consolidation time due to the slow water discharge rate, the water of the MSAs using CyanoMS was retained for a long period of up to 100 days. Thereafter, the water content decreased slowly. Given that no leaching liquid at the high WHC was observed in this study, most nutritional liquids originating from the CyanoMS may have been absorbed in the bulking agents of the MSAs. Thus, moisture content can be maintained at a proper level during composting of MSAs thus supporting a favorable environment for most soil microbes and nutrient development as shown **Fig. S1** and **S2**.

**Table S1**. Physicochemical characteristics and elemental composition of raw materials and final composted products of the control and microalgal soil ameliorants (MSAs) after 530 days of composting (Continued)

| Description | Raw materials | | | | | | Compost products | | | | |
| --- | --- | --- | --- | --- | --- | --- | --- | --- | --- | --- | --- |
|  | CyanoMS | MB_single culture_ | | Additive | | |  |  |  |  |  |
|  |  | GA | BGA | Sawdust | Oil cake | Perlite | Control | MSA_1_ | MSA_2_ | MSA_3_ | MSA_4_ |
| Moisture (%) | 82.6±1.1 | 5.3 | 3.7 | 9.7±3.7 | 14.9±10.6 | 3.2±1.9 | 53.4±5.5 | 48.4±3.2 | 40.3±2.9 | 41.9±4.4 | 55.2±2.3 |
| pH (-log{H^+^}) | N.A. | N.A. | N.A. | N.A. | N.A. | N.A. | 6.4±0.4 | 6.9±0.5 | 7.0±0.3 | 7.1±0.2 | 6.4±0.2 |
| ρ*_wb_* (kg m^-3^) | 961.1±123.8 | 586.6 | 552.6 | 121.5±38.2 | 272.3±122.5 | 114.5±20.6 | 228.5±25.3 | 190.3±9.3 | 182.0±4.6 | 192.0±22.3 | 315.8±28.8 |
| ρ*_db_* (kg m^-3^) | 166.3±17.9 | 555.4 | 531.9 | 108.3±30.1 | 218.9±75.5 | 110.5±18.3 | 105.4±8.0 | 98.2±7.0 | 108.7±5.2 | 110.8±9.5 | 141.0±9.2 |
| TS (%) of total wet mass | 17.4±1.1 | 94.7 | 96.3 | 90.3±3.7 | 85.1±10.6 | 96.8±1.9 | 46.6±5.5 | 51.6±3.2 | 59.7±2.9 | 58.1±4.4 | 44.8±2.3 |
| VS (%) of total dry mass | 46.8±0.9 | 78.4 | 83.7 | 84.4±3.2 | 80.2±11.5 | 0.7±0.1 | 67.3±1.5 | 76.9±4.1 | 73.3±3.8 | 73.6±2.5 | 61.6±5.0 |
| V_v_ (%) | 27.3±10.1 | 68.3 | 69.0 | 76.3±5.6 | 86.6±5.5 | 95.5±0.7 | 88.3±1.9 | 90.7±0.5 | 91.6±0.3 | 91.0±1.2 | 83.8±1.7 |
| V_a_ (%) | 13.6±11.5 | 65.4 | 67.0 | 84.4±3.2 | 82.6±8.9 | 95.2±1.0 | 82.7±2.4 | 86.0±0.7 | 87.2±0.4 | 86.4±1.9 | 76.0±2.4 |
| V_w_ (%) | 13.7±1.5 | 3.0 | 2.0 | 1.2±0.7 | 4.1±3.4 | 0.4±0.2 | 5.6±0.5 | 4.7±0.2 | 4.4±0.2 | 4.6±0.6 | 7.8±0.7 |
| WHC (g water/ g dry sample^-1^) | 7.33±0.00 | N.A. | N.A. | 1.37±0.07 | 2.30±0.26 | 1.52±0.03 | 3.26±0.27 | 3.05±0.15 | 3.97±0.01 | 2.93±0.09 | 2.47±0.17 |
| BET surface area (m^2^ g^-1^) | 18.97-144.34 | 20.68 | 16.74 | 0.19^a^ | 46.23 | 1.90^b^ | 1.91 | 1.40 | 1.64 | 4.18 | 4.08 |
| Total pore volume (cm^3^ g^-1^) | N.A. | N.A. | N.A. | N.A. | N.A. | N.A. | 9.5E-03 | 4.8E-03 | 6.4E-03 | 1.6E-02 | 1.8E-02 |
| Average pore diameter (nm) | N.A. | N.A. | N.A. | N.A. | N.A. | N.A. | 19.9 | 13.6 | 15.5 | 15.8 | 17.3 |
| Salinity (%) | N.A. | N.A. | N.A. | N.A. | N.A. | N.A. | 0.13±0.1 | 0.12±0.1 | 0.14±0.1 | 0.17±0.1 | 0.20±0.2 |
| HCl insoluble substance (%) | N.A. | N.A. | N.A. | N.A. | N.A. | N.A. | 14.35±0.71 | 6.69±0.85 | 7.01±0.74 | 8.95±0.77 | 11.07±0.85 |
| CEC (cmol kg^-1^) | N.A. | N.A. | N.A. | N.A. | N.A. | N.A. | 41.24±1.52 | 40.80±2.33 | 47.67±3.25 | 49.40±2.99 | 44.88±3.05 |

Notes: CyanoMS (n=1–5); MB_single culture_ means microalgal biomass as a single culture with green algae (GA) as *Chlorella* sp. (dry) (n=1) and blue green algae (BGA) as *Spirulina* sp. (dry) (n=1); Sawdust (n=1–4); Oil cake (n=1–4): Perlite (n=1–3); Control means no CyanoMS treatment (CyanoMS 0%) (n=1–5); MSA_1_ means microalgal soil ameliorants with CyanoMS treatment (CyanoMS 11.7%) (n=1–5); MSA_3_ means microalgal soil ameliorants with CyanoMS treatment (CyanoMS 21.6%) (n=1–5); MSA_3_ means microalgal soil ameliorants with CyanoMS treatment (CyanoMS 37.6%) (n=1–5); MSA_4_ means microalgal soil ameliorants with CyanoMS treatment (CyanoMS 59.5%) (n=1–5). N.D. means no data. ^a^ was referenced by Sh et al. (2020); ^b^ was referenced by Wheelwright et al. (2017).

**Table S1**. Continued from previous page

| Description | Raw materials | | | | | | Compost products | | | | |
| --- | --- | --- | --- | --- | --- | --- | --- | --- | --- | --- | --- |
|  | CyanoMS | MB_single culture_ | | Additive | | |  |  |  |  |  |
|  |  | GA | BGA | Sawdust | Oil cake | Perlite | Control | MSA_1_ | MSA_2_ | MSA_3_ | MSA_4_ |
| C/N ratio | 9.58 | 4.89 | 4.31 | 186.30 | 7.20 | 8.50 | 23.55 | 4.07 | 6.40 | 5.91 | 6.35 |
| Carbon, C (%) | 40.61 | 46.65 | 44.00 | 42.85 | 46.60 | 0.17 | 14.60 | 14.34 | 24.50 | 22.65 | 13.20 |
| Oxygen, O (%) | N.A. | N.A. | N.A. | N.A. | N.A. | N.A. | 50.09 | 16.80 | 25.02 | 24.18 | 23.32 |
| Nitrogen, N (%) | 4.24 | 9.54 | 10.2 | 0.23 | 6.47 | 0.02 | 0.62 | 3.52 | 3.83 | 3.83 | 2.08 |
| Sulfur, S (%) | 1.04 | 0.70 | 0.84 | 0.54 | 0.71 | 0.52 | 0.55 | 0.58 | 0.67 | 0.59 | 0.60 |
| Phosphorus, P (%) | 0.32 | 1.54 | 1.02 | 9.5E-03 | 1.49 | N.D. | 0.18 | 0.71 | 1.25 | 0.98 | 0.57 |
| Potassium, K (%) | 2.13 | 0.57 | 1.31 | 0.11 | 0.94 | N.D. | 0.26 | 0.60 | 0.86 | 0.71 | 0.54 |
| Iron, Fe (%) | 6.5E-03 | 2.9E-02 | 4.9E-02 | 2.5E-02 | 1.1E-02 | 6.4E-03 | 8.8E-02 | 4.2E-02 | 0.12 | 0.16 | 0.17 |
| Calcium, Ca (%) | 3.92 | 0.22 | 0.11 | 0.44 | 0.70 | 0.15E-03 | 0.71 | 0.47 | 0.57 | 0.51 | 0.33 |
| Magnesium, Mg (%) | 0.48 | 0.33 | 0.26 | 0.036 | 0.49 | 0.29E-02 | 0.13 | 0.28 | 0.35 | 0.31 | 0.28 |
| Aluminum, Al (%) | 1.11-1.85 | 0.02 | N.D. | 0.02 | N.D. | 0.21 | 0.11 | 0.16 | 0.38 | 0.33 | 0.29 |
| Manganese, Mn (%) | 5.9E-03 | 1.1E-03 | 1.0E-02 | 6.0E-03 | 8.5E-03 | 2.0E-04 | 1.4E-02 | 1.1E-02 | 1.6E-02 | 1.4E-02 | 9.4E-03 |
| Sodium, Na (%) | 4.0E-03 | 1.7E-02 | 1.28 | 7.6E-03 | 4.6E-03 | 0.16 | 1.8E-02 | 2.1E-02 | 2.3E-02 | 2.0E-02 | 2.4E-02 |
| Chloride, Cl (%) | N.D. | 4.6E-02 | 0.211 | 0.053 | 0.036 | 4.7E-02 | 4.8E-02 | 4.0E-02 | 4.2E-02 | 4.3E-02 | 4.5E-02 |
| Copper, Cu (%) | 9.0E-04 | N.D. | N.D. | N.D. | 2.6E-03 | N.D. | 4.6E-04 | 1.3E-03 | 2.5E-03 | 4.1E-03 | 5.3E-03 |
| Arsenic, As (%) | N.D. | N.D. | N.D. | N.D. | N.D. | N.D. | N.D. | N.D. | N.D. | N.D. | N.D. |
| Cadmium, Cd (%) | N.D. | N.D. | N.D. | N.D. | N.D. | N.D. | N.D. | N.D. | N.D. | N.D. | N.D. |
| Chromium, Cr (%) | N.D. | N.D. | N.D. | N.D. | N.D. | N.D. | N.D. | N.D. | N.D. | 3.4E-04 | 3.9E-04 |
| Mercury, Hg (%) | N.D. | 6.1E-06 | 7.2E-06 | 6.8E-06 | 6.2E-06 | N.D. | 3.2E-06 | 3.4E-06 | 1.7E-06 | 4.1E-06 | 1.6E-06 |

Notes: CyanoMS (n=1–5); MB_single culture_ means microalgal biomass as a single culture with green algae (GA) as *Chlorella* sp. (dry) (n=1) and blue green algae (BGA) as *Spirulina* sp. (dry) (n=1); Sawdust (n=1–4); Oil cake (n=1–4): Perlite (n=1–3); Control means no CyanoMS treatment (CyanoMS 0%) (n=1–5); MSA_1_ means microalgal soil ameliorants with CyanoMS treatment (CyanoMS 11.7%) (n=1–5); MSA_3_ means microalgal soil ameliorants with CyanoMS treatment (CyanoMS 21.6%) (n=1–5); MSA_3_ means microalgal soil ameliorants with CyanoMS treatment (CyanoMS 37.6%) (n=1–5); MSA_4_ means microalgal soil ameliorants with CyanoMS treatment (CyanoMS 59.5%) (n=1–5). N.D. means no data.

**Table S2**. Water contents, C/N ratio, and organic matter (OM) changes of microalgal soil ameliorants (MSAs) during composting process

| Compost products | Time (days) | Water contents (%) | C/N ratio | OM (%) |
| --- | --- | --- | --- | --- |
| Control | 1 | 69.0 | 109.1 | 29.7 |
|  | 31 | 51.5 | 56.5 | 36.7 |
|  | 52 | 44.7 | 39.9 | 38.8 |
|  | 83 | 49.9 | 34.1 | 38.8 |
|  | 102 | 58.1 | 32.6 | 32.9 |
|  | 126 | 55.4 | 32.2 | 30.5 |
|  | 225 | 59.8 | 28.1 | 29.2 |
|  | 530 | 63.4 | 23.6 | 39.5 |
| MSA_1_ | 1 | 58.2 | 36.3 | 46.2 |
|  | 31 | 61.5 | 30.8 | 36.0 |
|  | 52 | 72.3 | 22.9 | 23.9 |
|  | 83 | 72.1 | 12.9 | 24.4 |
|  | 102 | 72.2 | 17.8 | 22.4 |
|  | 126 | 72.2 | 19.8 | 23.7 |
|  | 225 | 68.1 | 16.5 | 21.5 |
|  | 530 | 48.4 | 4.1 | 27.9 |
| MSA_2_ | 1 | 59.2 | 39.2 | 46.9 |
|  | 31 | 53.6 | 29.1 | 41.6 |
|  | 52 | 62.6 | 21.3 | 32.0 |
|  | 83 | 68.2 | 14.1 | 26.6 |
|  | 102 | 68.3 | 16.1 | 24.6 |
|  | 126 | 68.3 | 17.5 | 27.0 |
|  | 225 | 66.8 | 15.4 | 25.7 |
|  | 530 | 40.3 | 6.4 | 22.8 |
| MSA_3_ | 1 | 60.7 | 32.6 | 44.4 |
|  | 31 | 63.6 | 26.7 | 31.2 |
|  | 52 | 67.1 | 20.0 | 27.7 |
|  | 83 | 70.8 | 18.7 | 24.9 |
|  | 102 | 69.1 | 19.0 | 24.1 |
|  | 126 | 69.8 | 19.8 | 24.9 |
|  | 225 | 67.9 | 16.2 | 24.4 |
|  | 530 | 41.9 | 5.9 | 25.3 |
| MSA_4_ | 1 | 68.0 | 26.4 | 37.9 |
|  | 31 | 72.2 | 21.2 | 23.5 |
|  | 52 | 74.8 | 21.6 | 20.0 |
|  | 83 | 75.1 | 22.2 | 19.3 |
|  | 102 | 75.1 | 18.7 | 18.2 |
|  | 126 | 75.4 | 19.3 | 17.8 |
|  | 225 | 74.6 | 17.7 | 17.1 |
|  | 530 | 55.2 | 6.4 | 13.1 |

**Fig. S1**. Long-term monitoring results of the macro-nutrients and micro-nutrients release of the microalgal soil ameliorants (MSAs). (a) TN. (b) NH_4_^+^-N. (c) P_2_O_5_. (d) K_2_O. (e) MgO. (f) CaO.

**Fig. S2**. Dependence of different total microbial population changes on composting time.

**References**

1. Brinton, W., 2000. Compost quality standards and guidelines. Final Report by Woods End Research Laboratories for the New York State Association of Recyclers.
2. Goličnik, M., 2018. Closed-form solution of the reduced Haldane equation for enzyme kinetics with strong substrate inhibition. Communications in mathematical and in. Comput. Chem. 79, 607–618.
3. Han, S., Li, J., Zhou, Q., Liu, G., Wang, T., 2019. Harmless disposal and resource utilization of wastes from the lake in China: Dewatering, composting and safety evaluation of fertilizer. Algal Res. 43. [101623](http://www.ncbi.nlm.nih.gov/pubmed/101623).
4. Huang, Y., Li, R., Liu, H., Wang, B., Zhang, C., Shen, Q., 2014. Novel resource utilization of refloated algal sludge to improve the quality of organic fertilizer. Environ. Technol. 35, 1658–1667.
5. Qian, Y., Zhu, W., Sun, Z., Gong, M., 2014. Experiment on consolidation properties of cyanobacterial. J. Water Resour. Water Eng. 25, 136–139.
6. Saha, J.K., Panwar, N., Singh, M.V., 2010. An assessment of municipal solid waste compost quality produced in different cities of India in the perspective of developing quality control indices. Waste Manag. 30, 192–201.
7. Sánchez, Ó.J., Ospina, D.A., Montoya, S., 2017. Compost supplementation with nutrients and microorganisms in composting process. Waste Manag. 69, 136–153.
8. Sh, L., Lee, B., Jeong, T., Jeon, C., 2020. Effects of different pretreatment methods on the grindability of pitch pine sawdust biomass and its blends with coal. J. Mech. Sci. Technol. 34, 2235–2243.
9. Wheelwright, W., Cooney, R.P., Ray, S., Zujovic, Z., de Silva, K., 2017. Ultra-high surface area nano-porous silica from expanded perlite: Formation and characterization. Ceram. Int. 43, 11495–11504.
10. Zaffar, M., Lu, S., 2015. Pore Size Distribution of Clayey Soils and Its Correlation with soil organic matter. Pedosphere 25, 240–249.
